# Prompt Framework for Extracting Scale-Related Knowledge Entities from Chinese Medical Literature: Development and Evaluation Study

**DOI:** 10.2196/67033

**Published:** 2025-03-18

**Authors:** Jie Hao, Zhenli Chen, Qinglong Peng, Liang Zhao, Wanqing Zhao, Shan Cong, Junlian Li, Jiao Li, Qing Qian, Haixia Sun

**Affiliations:** 1 Institute of Medical Information/Medical Library Chinese Academy of Medical Sciences & Peking Union Medical College Beijing China; 2 Qingdao Innovation and Development Center Harbin Engineering University Qingdao China; 3 College of Intelligent Systems Science and Engineering Harbin Engineering University Harbin China; 4 Department of Thoracic Surgery National Cancer Center/National Clinical Research Center for Cancer/Cancer Hospital Chinese Academy of Medical Sciences & Peking Union Medical College Beijing China

**Keywords:** prompt engineering, named entity recognition, in-context learning, large language model, Chinese medical literature, measurement-based care, framework, prompt, prompt framework, scale, China, medical literature, MBC, LLM, MedScaleNER, retrieval, information retrieval, dataset, artificial intelligence, AI

## Abstract

**Background:**

Measurement-based care improves patient outcomes by using standardized scales, but its widespread adoption is hindered by the lack of accessible and structured knowledge, particularly in unstructured Chinese medical literature. Extracting scale-related knowledge entities from these texts is challenging due to limited annotated data. While large language models (LLMs) show promise in named entity recognition (NER), specialized prompting strategies are needed to accurately recognize medical scale-related entities, especially in low-resource settings.

**Objective:**

This study aims to develop and evaluate MedScaleNER, a task-oriented prompt framework designed to optimize LLM performance in recognizing medical scale-related entities from Chinese medical literature.

**Methods:**

MedScaleNER incorporates demonstration retrieval within in-context learning, chain-of-thought prompting, and self-verification strategies to improve performance. The framework dynamically retrieves optimal examples using a k-nearest neighbors approach and decomposes the NER task into two subtasks: entity type identification and entity labeling. Self-verification ensures the reliability of the final output. A dataset of manually annotated Chinese medical journal papers was constructed, focusing on three key entity types: scale names, measurement concepts, and measurement items. Experiments were conducted by varying the number of examples and the proportion of training data to evaluate performance in low-resource settings. Additionally, MedScaleNER’s performance was compared with locally fine-tuned models.

**Results:**

The CMedS-NER (Chinese Medical Scale Corpus for Named Entity Recognition) dataset, containing 720 papers with 27,499 manually annotated scale-related knowledge entities, was used for evaluation. Initial experiments identified GLM-4-0520 as the best-performing LLM among six tested models. When applied with GLM-4-0520, MedScaleNER significantly improved NER performance for scale-related entities, achieving a macro *F*_1_-score of 59.64% in an exact string match with the full training dataset. The highest performance was achieved with 20-shot demonstrations. Under low-resource scenarios (eg, 1% of the training data), MedScaleNER outperformed all tested locally fine-tuned models. Ablation studies highlighted the importance of demonstration retrieval and self-verification in improving model reliability. Error analysis revealed four main types of mistakes: identification errors, type errors, boundary errors, and missing entities, indicating areas for further improvement.

**Conclusions:**

MedScaleNER advances the application of LLMs and prompts engineering for specialized NER tasks in Chinese medical literature. By addressing the challenges of unstructured texts and limited annotated data, MedScaleNER’s adaptability to various biomedical contexts supports more efficient and reliable knowledge extraction, contributing to broader measurement-based care implementation and improved clinical and research outcomes.

## Introduction

Measurement-based care (MBC), which involves the systematic use of standardized scales to assess patient conditions, track progress, and inform clinical decisions, has been shown to enhance patient outcomes and optimize health care processes [1]. Despite its significant benefits, MBC remains underused worldwide, with less than 20% of health practitioners incorporating it into their routine practice [2]. A primary barrier is the lack of accessible and comprehensive knowledge about these scales [3,4]. Clinicians are often unaware of which scales are suitable for specific conditions and lack a clear understanding of the concepts and items within those scales [5]. Acquiring this knowledge requires considerable time and effort in searching and reviewing various unstructured documents, such as technical reports, academic papers, and manuals. This not only adds to the workload of already busy medical professionals [6] but also hinders the widespread adoption of MBC [7]. Transforming unstructured scale-related documents into computable and accessible knowledge systems, such as knowledge graphs, could help alleviate the burden on practitioners and promote MBC adoption [8].

The key to this transformation lies in accurately extracting scale-related knowledge entities from unstructured medical texts. However, several challenges complicate this task. The complexity of medical language, coupled with the variety of scale-related entities, including scale names, measurement concepts, and measurement items, makes accurate extraction difficult [1]. For instance, the entity “scale name” may refer solely to the scale itself or include additional details such as its developer, language, version, or population-specific characteristics. Furthermore, extracting knowledge entities from Chinese medical texts introduces additional difficulties due to linguistic variations and the limited availability of annotated data specific to medical scales in Chinese [9]. Traditional information extraction methods often depend on extensive data annotation and model fine-tuning, which are resource-intensive and struggle to adapt to new tasks or domains.

Addressing these challenges requires innovative solutions that can handle the complexity and variability of medical scale information, particularly in Chinese, with limited resources. Large language models (LLMs) such as GPT [10], GLM [11], LLaMA [12], and Qwen [13] have recently demonstrated impressive capabilities in natural language understanding and in-context learning (ICL) [14], enabling them to perform complex language tasks with minimal fine-tuning and limited annotated data [15,16]. By leveraging prompt engineering, these models can generate targeted outputs from well-crafted inputs, reducing the need for extensive datasets and making them particularly valuable in low-resource scenarios [17-19]. Moreover, LLMs have shown the ability to perform tasks in zero-shot and few-shot settings [20,21], allowing them to generalize from minimal demonstrations (examples) [22]. However, their effectiveness in information extraction tasks is highly dependent on the quality of the prompt design, including the task instructions, example selection, and output formats [23]. In few-shot settings, selecting the most appropriate examples is crucial for guiding the LLM toward accurate information extraction [24]. This selection process is particularly challenging in specialized domains, where the examples should not only be relevant but also representative of the task’s complexity.

To improve the performance of LLMs in information extraction tasks, researchers have explored techniques such as demonstration retrieval within the ICL paradigm [25]. In this approach, relevant examples are dynamically selected from a pool of annotated data based on their similarity to the input text, addressing the issue of sample representativeness [26]. Moreover, techniques such as chain-of-thought (CoT) prompting [27] and self-verification [25] have been used to enhance the accuracy and robustness of domain-specific tasks. CoT prompting involves breaking down complex tasks into subtasks, and guiding the LLM step-by-step through multiturn dialogue [27], while self-verification allows the model to review and refine its outputs [28]. Despite these promising strategies, most existing research focuses on extracting common medical entities such as diseases, symptoms, drugs, and procedures [29], with limited attention to the extraction of scale-related entities.

Given the importance of task-oriented prompt design and the linguistic complexities of Chinese medical literature, there is an urgent need to develop a specialized framework for medical scale–related knowledge extraction. In this study, we propose MedScaleNER, a task-oriented prompt framework tailored for named entity recognition (NER) of medical scales in Chinese medical literature. MedScaleNER incorporates demonstration retrieval, CoT prompting, and self-verification strategies to tackle the specific challenges associated with extracting scale-related knowledge entities in Chinese. By dynamically selecting representative examples, the framework enhances the generalization capabilities of LLMs and improves extraction performance in few-shot scenarios. CoT prompting decomposes the scale-related NER task into manageable subtasks, easing the cognitive load on LLMs, while self-verification ensures output reliability.

This study emphasizes the significance of prompt design in LLM-based information extraction, particularly in specialized domains with limited annotated data. By combining demonstration retrieval with advanced prompt strategies, we aim to overcome the challenges posed by data scarcity and the linguistic variations of Chinese medical literature. To facilitate evaluation, we constructed a manually annotated corpus of Chinese medical scales, covering three key entity types of scale names, measurement concepts, and measurement items. We conducted an in-depth assessment of MedScaleNER’s effectiveness on this self-built dataset, examining the impact of the number of demonstrations, the contributions of CoT and self-verification, and the annotated data size required for optimal performance. Our approach contributes to building comprehensive scale knowledge systems, supporting clinicians and researchers in clinical and research efforts, promoting MBC adoption, and ultimately improving patient care.

## Methods

### Overview

**Figure 1:**
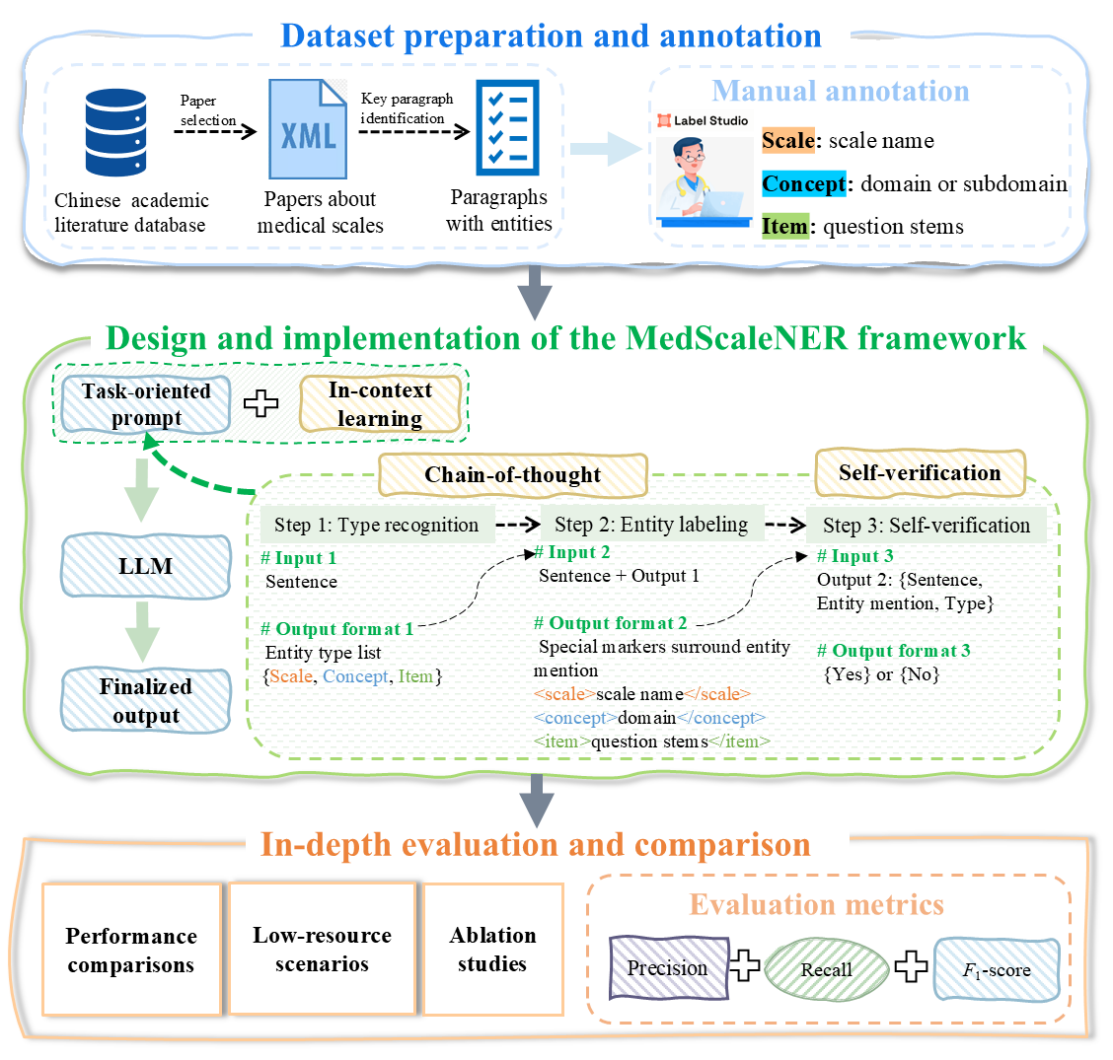
Workflow of the MedScaleNER prompt framework. LLM: large language model.

The workflow of the proposed MedScaleNER prompt framework is illustrated in Figure 1 and consists of three main stages: dataset preparation and annotation, design and implementation of the MedScaleNER framework, and in-depth evaluation and comparison. The process begins with the collection of high-quality Chinese journal papers focused on medical scales. These papers are preprocessed and manually annotated to extract three key types of scale-related entities: scale names, measurement concepts, and measurement items. This manually annotated corpus fills the gap caused by the limited availability of annotated data in this area, while also reflecting the complexities unique to Chinese medical literature.

To address the task of medical scale–related NER, we introduce the MedScaleNER framework, which incorporates demonstration retrieval within the ICL paradigm, CoT prompting, and self-verification techniques. The framework selects relevant examples dynamically, breaks down complex NER tasks into manageable subtasks, and improves the reliability of outputs through self-verification. We evaluate MedScaleNER comprehensively, including comparisons of performance with varying numbers of retrieved demonstrations, ablation studies to determine the impact of CoT and self-verification, and assessments of its effectiveness in low-resource scenarios with different training data sizes. Additionally, we benchmark its performance against traditional fine-tuned LLMs in local.

Formally, the task is defined as follows. Given a collection of Chinese academic documents related to medical scales, denoted as *D*, where each document *D_i_* consists of a sequence of sentences *S* = {*s_1_*, *s_2_*, ..., *s_n_*}, and a set of entity types *T* = {scale, concept, item}, the goal of MedScaleNER is to identify all entities *e_i_* within *D* and assign the appropriate type *t_i_* ∈ *T* to each identified entity.

### Ethical Considerations

This study used only publicly available published papers from the China National Knowledge Infrastructure, which consist of academic literature and do not contain real patient information. Since the data is publicly accessible and does not involve human participants or private data, ethical approval was not required.

### Dataset Preparation and Annotation

Due to the lack of annotated datasets for knowledge entity recognition in Chinese medical scales, we constructed a manually annotated corpus from full-text medical journal papers. The annotation focused on three key types of knowledge entities within medical scales: scale name, measurement concept, and measurement item. The scale name refers to the official or widely recognized title of the medical scale used in MBC, such as “The M. D. Anderson Symptom Inventory.” The measurement concept is defined as the broader theoretical or clinical construct that the scale is designed to assess, such as anxiety or cognitive function. The measurement item, on the other hand, refers to the individual questions within the scale that evaluate specific aspects of the measurement concept.

We began by retrieving abstracts of Chinese core medical journal papers from the China National Knowledge Infrastructure [30], which is a Chinese academic journal full-text database, targeting scale development research. The search was conducted using the “Abstract” and “Chinese Library Classification” criteria. From the retrieved papers, we selected the top three subfields within the Chinese Library Classification R code (Medicine and Health) based on literature frequency. Each abstract was manually reviewed to ensure the inclusion of original research papers, and the corresponding full texts were obtained in XML format. A detailed analysis of these full texts revealed that the Methods, Results, and Discussion sections contained a higher density of mentions related to scale names, measurement concepts, and items. Compared to scale names, mentions of concepts and items were less frequent, with items being particularly sparse.

To improve the balance and density of these entities, we extracted paragraphs specifically from the Methods, Results, and Discussion sections based on their XML structure. We then used key clue words such as “dimension,” “domain,” “variable,” “concept,” “factor,” “item,” and “entry” to identify paragraphs likely to contain the targeted entities. Paragraphs containing these terms were retained for annotation, while others were excluded.

For data annotation, we used the Label Studio tool [31]. Prior to formal annotation, a preannotation phase was conducted to train annotators. During this phase, annotators were introduced to the annotation scheme, guidelines (summarized in Multimedia Appendix 1), and tools. Feedback from this stage was used to refine both the scheme and guidelines through discussions. In the formal annotation phase, each paper was independently annotated by two annotators. A third annotator then checked for consistency, corrected discrepancies based on either annotator’s results, addressed missed annotations, and documented uncertain cases, which were later resolved through group discussions. Cohen κ coefficient was calculated to assess annotation consistency, yielding an overall score of 0.95, indicating a high level of reliability for the constructed dataset [32]. Specifically, the type-specific Cohen κ values were 0.961 for scale entities, 0.950 for concepts, and 0.970 for items.

### Design and Implementation of the MedScaleNER Framework

#### Overview

**Figure 2:**
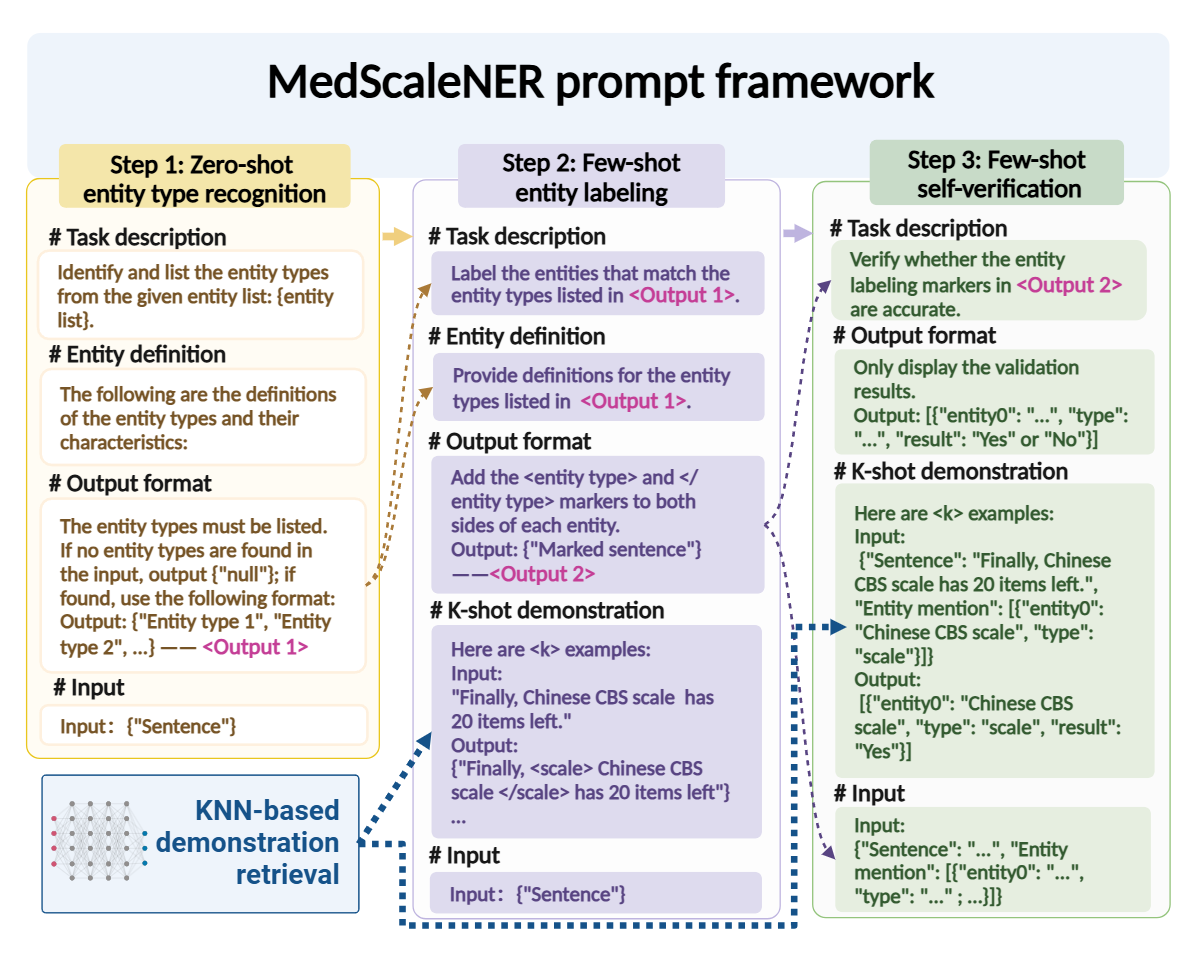
Design of the MedScaleNER prompt framework. KNN: k-nearest neighbors.

We developed the MedScaleNER prompt framework to identify scale-related entities in medical texts using LLMs. The framework is designed to optimize entity recognition by incorporating three key stages: zero-shot entity type recognition, few-shot entity labeling, and self-verification. To enhance LLM performance, MedScaleNER integrates CoT prompting, which helps guide the model step by step through complex tasks, reducing the cognitive load. This is achieved by first identifying entity types in a zero-shot setting and then labeling the entities with a few examples. To further improve contextual understanding, the framework dynamically retrieves relevant examples using k-nearest neighbors (KNN) and uses self-verification to minimize hallucinations and overprediction, which are common issues in NER tasks [33]. Figure 2 outlines the MedScaleNER prompt framework, which consists of four main components: demonstration retrieval, entity type recognition, entity labeling, and self-verification.

#### Step 1: Zero-Shot Entity Type Recognition

In the first step, we design a prompt that includes four essential components: task description, entity definitions, output format, and input text [25]. Previous research in medical entity recognition using LLMs emphasizes the critical importance of prompt design, especially in specialized tasks [34]. Therefore, in this step, the model is framed as a “sophisticated linguist and named entity annotation expert” and tasked with identifying and listing entity types present in the input text without examples. This is the zero-shot stage, where the LLM uses only the provided definitions to perform entity-type recognition without any prior labeled examples.

To improve the model’s understanding of domain-specific terminology, we provide clear definitions and possible forms for each entity type. This enables the LLM to comprehend and distinguish between different medical scale–related entities. The output is structured as a list of identified entity types, with explicit instructions to avoid unnecessary information, limit responses to the provided entity list, and return “{null}” if no entities are found. The output is structured as a list of identified entity types present in the input text, without repeating types for multiple occurrences. For instance, if the entity type “scale” appears multiple times in the input, it is represented only once in the output to indicate its presence. Step 1 is instructed to limit responses to the provided entity type list and return “{null}” if no entities are found. By guiding the model through these structured prompts, we leverage CoT prompting to break down the task into manageable steps for better performance (detailed in Multimedia Appendix 2).

#### Step 2: Few-Shot Entity Labeling

The second step builds upon the output of step 1 by introducing few-shot entity labeling. The prompt in this stage includes the task description, entity definitions, examples (demonstrations), output format, and the input sentence. The role of the LLM remains that of a named entity annotation expert. Now, the task is to label the entities that match the entity types identified in step 1 within the input text.

**Figure 3:**
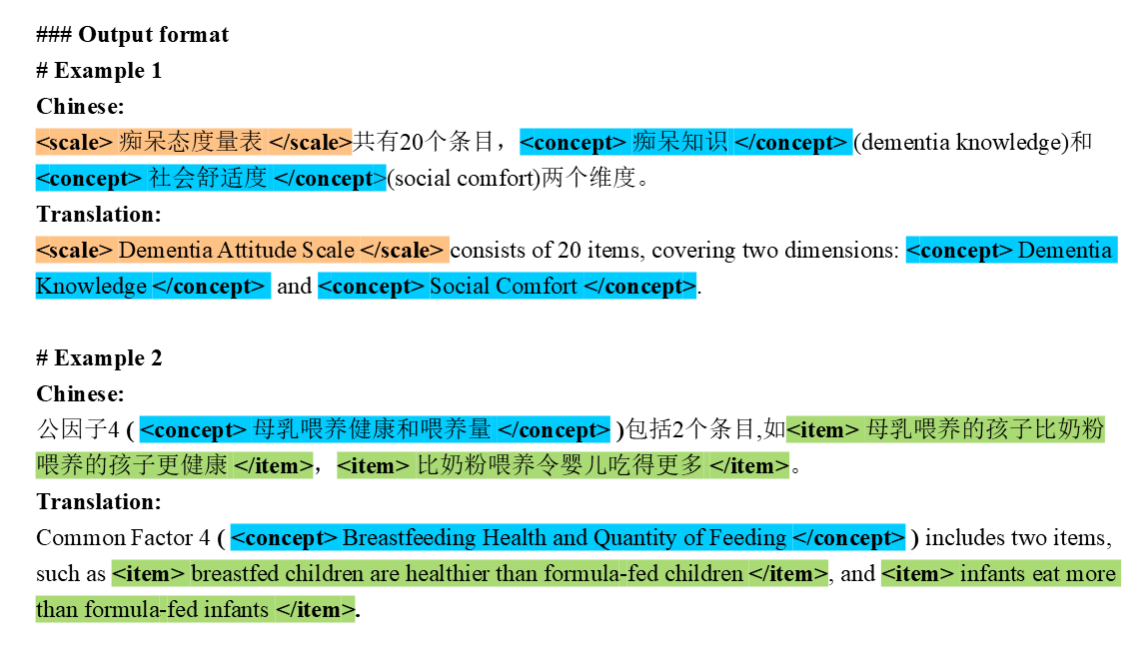
Output format of entity labeling.

Here, we use few-shot prompting because providing a small number of high-quality examples typically boosts performance, especially in specialized tasks like medical NER [35]. Few-shot prompting often achieves results comparable to those of fine-tuned models trained on hundreds of samples. However, it is sensitive to the representativeness of the examples, as well as the length of the input. To mitigate these challenges, we use KNN retrieval to dynamically select the most relevant examples from the training corpus. These examples, which are semantically similar to the input text, serve as demonstrations for the LLM to follow, guiding it in accurately labeling entities within the text. Moreover, we incorporate CoT prompting by breaking the task into incremental steps: first identifying entity types (step 1), followed by entity labeling (step 2). Step 1 involves identifying the entity types present in the input text, which informs the candidate pool for KNN retrieval in step 2. For instance, if step 1 determines that the entity types are {scale, concept}, step 2 specifically retrieves examples containing both scale and concept entities. The LLM surrounds the identified entities with appropriate markers in the text [36], as illustrated in Figure 3, with detailed prompts provided in Multimedia Appendix 3.

#### KNN Demonstration Retrieval

For demonstration retrieval, we use KNN, a widely used method in ICL [37]. The KNN algorithm retrieves semantically similar examples from the annotated dataset to provide the LLM with contextually relevant demonstrations. We begin by generating sentence-level embeddings for both the input texts and candidate examples using the SimCSE [38] method implemented in the acge_text_embedding model [39]. The cosine similarity between the input text and each candidate example is then computed, and the top k examples with the highest similarity scores are selected.

To enable efficient retrieval from large-scale, high-dimensional embedding spaces, we use the FAISS library [40]. FAISS provides specialized data structures and algorithms for fast indexing and searching of embeddings. After indexing the training embeddings, retrieving demonstrations for a given test sentence simply involves extracting its embedding and performing a semantic similarity search against the indexed embeddings. This approach significantly reduces computational overhead by eliminating exhaustive pairwise comparisons.

To determine the optimal value of *k*, we conducted experiments with a range of different *k* values. We selected the value that maximized model performance by balancing the need for enough examples to help the LLM generalize while avoiding performance degradation caused by irrelevant or excessive examples. These examples are fed to the LLM to improve generalization and entity labeling.

#### Step 3: Few-Shot Self-Verification

The final step incorporates a self-verification mechanism to improve the accuracy and reliability of the labeled entities. After completing the entity labeling, the LLM undergoes a self-verification process through few-shot prompting, where it reviews its own output for correctness. In this step, the model’s task in this step is to verify whether the labeled entities are accurate by responding with either “Yes” or “No” for each entity. If the answer is “No,” the entity is removed from the output.

The input for this step includes both the labeled entities and their surrounding context, allowing the LLM to validate its previous output holistically. This self-verification step improves the reliability of the final results, ensuring that the identified and labeled entities meet the expected standards of accuracy (detailed prompts are provided in Multimedia Appendix 4).

### In-Depth Evaluation and Comparison

To comprehensively assess the performance of MedScaleNER, we conducted an in-depth analysis. Before the formal experiments, we first identified the best-performing LLM for use in MedScaleNER by comparing various LLMs accessed via the application programming interface (API). Following this, we compared MedScaleNER’s performance with that of locally fine-tuned LLMs on the NER task. Additionally, we carried out ablation studies focusing on the two key components of MedScaleNER: CoT prompting in step 1 (zero-shot entity type recognition) and self-verification in step 3. By isolating these components, we examined their individual contributions to the overall framework, specifically their impact on entity recognition accuracy and output robustness. These studies provided valuable insights into the importance of each step in enhancing model reliability and precision.

Furthermore, we evaluated MedScaleNER in low-resource settings by varying the amount of training data and the number of demonstrations in the few-shot setting (steps 2 and 3). This analysis was essential for understanding how the framework performs under limited data conditions and testing its scalability and effectiveness when annotation resources are scarce. By experimenting with different proportions of available data and examples, we gained insights into the adaptability of MedScaleNER in resource-constrained scenarios.

For evaluation, we used precision, recall, and macro *F*_1_-score. Precision represents the proportion of correctly predicted entities out of all entities predicted by the model. Recall is the proportion of correctly predicted entities out of all actual entities present in the dataset. Macro *F*_1_-score is the harmonic mean of precision and recall, averaged across all entity classes to account for imbalanced class distributions. We determined the correctness of entity recognition using exact string matching, meaning only perfect matches between the model’s predictions and the ground truth were considered correct. This strict evaluation method ensured a high standard for assessing model performance across all comparisons, providing a clear and objective measure of MedScaleNER’s effectiveness.

## Results

### Summary Statistics of CMedS-NER

We constructed the CMedS-NER (Chinese Medical Scale Corpus for Named Entity Recognition) dataset specifically for the NER task in the context of Chinese medical scales. The dataset consists of 720 full-text Chinese academic papers focused on medical scales, which include 5582 paragraphs and 22,743 sentences. After conducting a concordance test and making necessary emendations, CMedS-NER contained a total of 27,499 entity mentions. These consisted of 12,340 mentions of scales, 11,968 mentions of concepts, and 3191 mentions of items. For evaluation purposes, the dataset was randomly split at the document level into 90% for training and 10% for testing. Detailed characteristic statistics of the training and test data are presented in Table 1.

**Table 1:** Statistics of training and test data.

Data type		Training set, n (%)	Test set, n (%)	Total, n (%)
Papers		648 (90)	72 (10)	720 (100)
Paragraphs		5055 (90.56)	527 (9.44)	5582 (100)
Sentences		20,454 (89.94)	2289 (10.06)	22,743 (100)
**Entities**				
	Scale	11,106 (90)	1234 (10)	12,340 (100)
	Concept	10,836 (90.54)	1132 (9.46)	11,968 (100)
	Item	2947 (92.35)	244 (7.65)	3191 (100)
	All	24,889 (90.51)	2610 (9.49)	27,499 (100)

### LLM Selection and Experimental Setup

To determine the best-performing LLM for the MedScaleNER framework, we conducted preliminary experiments with six generative LLMs: GPT-3.5-turbo, GLM-4-0520, ERNIE-Bot-turbo, Moonshot-v1-8k, AGI Sky-Chat-3.0, and Qwen-turbo-0624. These models were accessed via APIs and evaluated on randomly selected sentences from the CMedS-NER test set, which included ten scale entities. Among the tested models, GLM-4-0520 performed the best, accurately recognizing nine out of ten scale entities, Qwen-turbo-0624 followed, identifying eight entities (complete results are provided in Multimedia Appendix 5). Based on this superior performance, GLM-4-0520 was selected for subsequent experiments. For the GLM-4-0520 setup, we used temperature sampling, setting the temperature parameter to 0.02 and the max_tokens parameter to 2048, while leaving all other hyperparameters at their default values.

In addition to evaluating the API-accessed LLM, we implemented local fine-tuning for four models: GLM-4-9B-Chat [41], Qwen2-7B [42], BiLSTM-CRF [43] (Chinese-BERT-wwm), and W2NER [44] (MacBERT). Fine-tuning was performed using Pytorch 1.12.1+cu11.6 on NVIDIA RTX A6000 graphics processing units. For these locally fine-tuned models, hyperparameters were optimized using empirical tuning methods to achieve the best performance on the CMedS-NER dataset. Detailed hyperparameter settings for each model are provided in Multimedia Appendix 6.

### Performance Comparisons

#### Optimal k-Shot Demonstration Selection

To determine the optimal number of demonstrations (k) for few-shot learning, we tested various k-shot settings (0, 5, 10, 15, 20, and 25) on a randomly selected set of 100 sentences from the test set. As shown in Table 2, overall performance improved as the number of demonstrations increased, with the highest *F*_1_-score of 81.23% achieved at 20-shot. However, different entity types peaked at different k values. For instance, the *F*_1_-score for concepts peaked at 10-shot (85.07%), while the *F*_1_-score for scales and items reached their highest performance at 20-shot, with scores of 77.27% and 84.21%, respectively.

**Table 2:** Entity extraction performance with different k-shot values.

k-shot	All, *F*_1_-score (%)	Scale, *F*_1_-score (%)	Concept, *F*_1_-score (%)	Item, *F*_1_-score (%)
0	17.11	23.26	28.07	0
5	68.74	61.87	74.34	70
10	67.59	67.69	*85.07* ^a^	50
15	78.55	71.88	83.78	80
20	*81.23*	*77.27*	82.19	*84.21*
25	71.06	75.19	78.73	59.26

^a^The best performance is italicized.

#### Low-Resource Comparison

**Figure 4:**
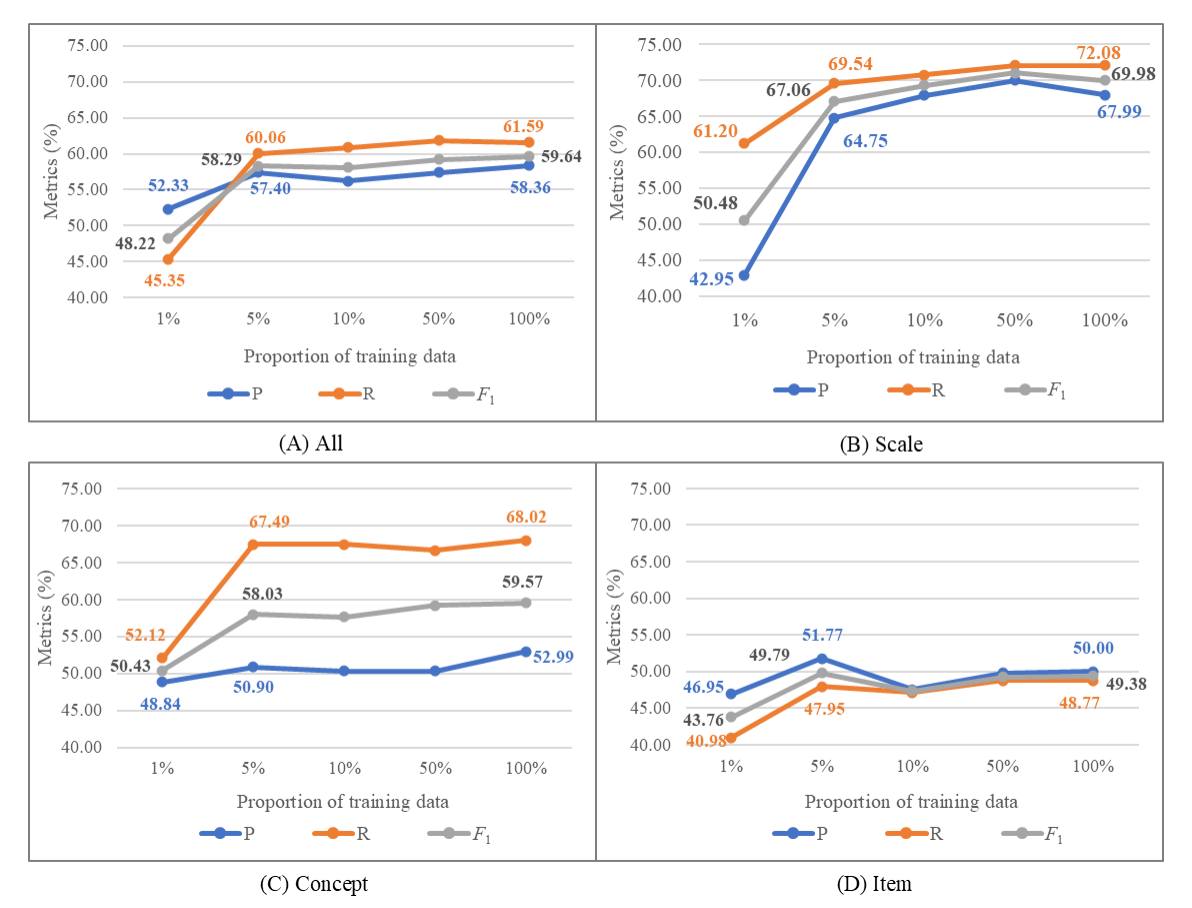
Low-resource performance of MedScaleNER: precision (P), recall (R), and macro *F*_1_-scores (*F*_1_) across different proportions of training data for (A) all entities, (B) scales, (C) concepts, and (D) items.

To evaluate MedScaleNER’s performance in low-resource scenarios, we trained the model using different proportions of the training data (1%, 5%, 10%, 50%, and 100%) and assessed its performance on the test set. As presented in Figure 4A, the overall *F*_1_-score increased as more training data was used. Notably, significant performance gains were observed when increasing the training data from 1% (205 sentences) to 5% (1023 sentences), with the overall *F*_1_-score rising from 48.22% to 58.29%, precision improving from 52.33% to 57.40%, and recall jumping from 45.35% to 60.06%. Beyond this point, improvements plateaued, with only a 1.35% increase in the *F*_1_-score between 5% and 100% of the training data (from 58.29% to 59.64%). A similar trend was observed for precision and recall, although precision dropped slightly at 10% of the training data. When examining scale and concept entities (Figure 4B and C), the same pattern emerged: a significant improvement from 1% to 5% of the training data, followed by minimal gains from 5% to 100%. However, for item entities (Figure 4D), precision, recall, and *F*_1_-scores slightly declined as the training data increased from 5% to 100%.

#### Ablation Studies

To evaluate the contributions of different components within the MedScaleNER framework, we conducted ablation studies to examine the impact of CoT prompting and self-verification under various low-resource settings. We tested the GLM-4-0520’s performance with and without these components using different proportions of the training data (1%, 5%, 10%, 50%, and 100%). The baseline model involved directly prompting the task description and labeling entities in the input using 20 examples.

As shown in Table 3, incorporating self-verification consistently improved *F*_1_-scores across all training data sizes. With 100% of the training data, self-verification led to a 0.89% increase in the *F*_1_-score, while breaking down the task into subtasks using CoT resulted in a 2.11% increase. However, in extremely low-resource scenarios (eg, 1% of the training data), adding CoT without self-verification did not enhance performance and slightly decreased the *F*_1_-score. The combination of both CoT and self-verification yielded the best performance across all training data sizes. In low-resource settings, self-verification had a significant impact. It increased the *F*_1_-score of MedScaleNER by 7.92% with 1% of the training data and by 3.27% with 5% of the training data, compared to configurations without it (ie, baseline + CoT).

**Table 3:** Ablation study results: P^a^, R^b^, and *F*_1_^c^ scores for different components of MedScaleNER across different proportions of training data.

Proportion of training data (n of sentences)	Baseline				Baseline + self-verification				Baseline+ CoT^d^				MedScaleNER		
	P (%)	R (%)	*F*_1_ (%)	P (%)		R (%)	*F*_1_ (%)	P (%)		R (%)	*F*_1_ (%)	P (%)		R (%)	*F*_1_ (%)
1% (205)	48.21	45.70	46.63	51.42		45.59	48.14	36.52		45.46	40.30	52.33		45.35	48.22
5% (1023)	44.21	66.41	52.35	47.61		66.71	55.07	51.10		60.20	55.02	57.40		60.06	58.29
10% (2045)	45.88	66.11	54.37	47.99		65.54	55.03	50.84		60.95	55.13	56.22		60.84	58.11
50% (10,227)	45.12	68.08	54.32	48.86		68.41	56.76	52.49		62.16	56.69	57.41		61.83	59.23
100% (20,454)	45.21	69.50	54.78	47.79		69.31	55.67	53.11		61.73	56.89	58.36		61.59	59.64

^a^P: precision.

^b^R: recall.

^c^*F*_1_: macro *F*_1_-score.

^d^CoT: chain-of-thought.

#### Comparison With Local Fine-Tuned Models

We compared MedScaleNER with several locally fine-tuned models on the CMedS-NER dataset, including GLM-4-9B-Chat, Qwen2-7B, BiLSTM-CRF (Chinese-BERT-wwm), and W2NER (MacBERT). Both GLM-4-9B-Chat and Qwen2-7B were fine-tuned using the low-rank adaptation method with a parameter-efficient fine-tuning strategy with identical fine-tuning parameters, ensuring a fair comparison. After fine-tuning, we prompted the fine-tuned GLM-4-9B-Chat and Qwen2-7B for the NER task using a similar prompt structure as our Baseline, but without KNN retrieval.

**Figure 5:**
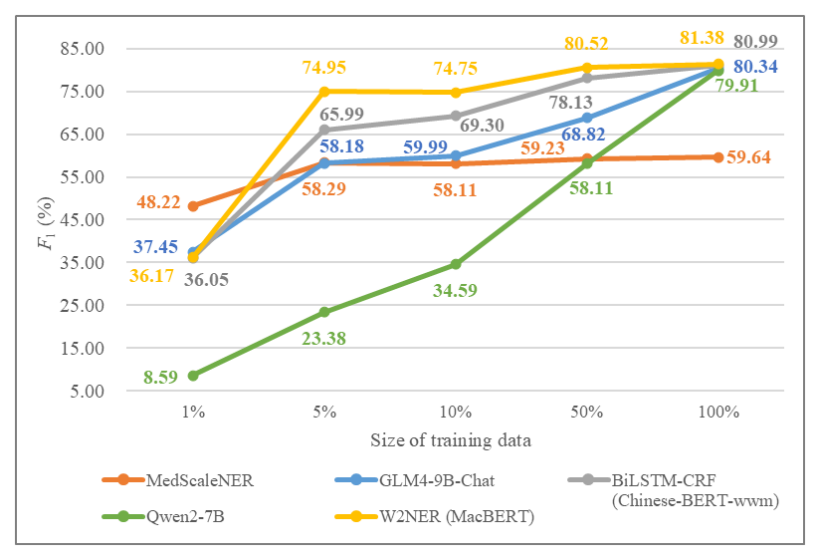
*F*_1_-score comparison of MedScaleNER and locally fine-tuned models across different proportions of training data.

As shown in Figure 5, MedScaleNER achieved an overall *F*_1_-score of 59.64%, which is lower than the fine-tuned Qwen2-7B (79.91%), GLM-4-9B-Chat (80.34%), BiLSTM-CRF (80.99%), and W2NER (81.38%). Notably, under low-resource scenarios (eg, using only 1% of the training data), MedScaleNER significantly outperformed the other fine-tuned models. At 5% of the training data, while MedScaleNER’s *F*_1_-score was lower than W2NER and BiLSTM-CRF, it remained substantially higher than Qwen2-7B and stayed competitive with GLM-4-9B-Chat.

### Error Analysis

We conducted an error analysis by manually reviewing 300 randomly selected sentences from the model outputs to identify common types of mistakes and areas for improvement. The errors were classified into four main types: (1) identification errors, where nonentity terms were incorrectly identified as entities; (2) type errors, where entities were correctly identified but assigned the wrong entity type; (3) boundary errors, which involved incorrect determination of the start and end positions of entities; and (4) missing entities, where entities present in the text were not identified by the model.

**Figure 6:**
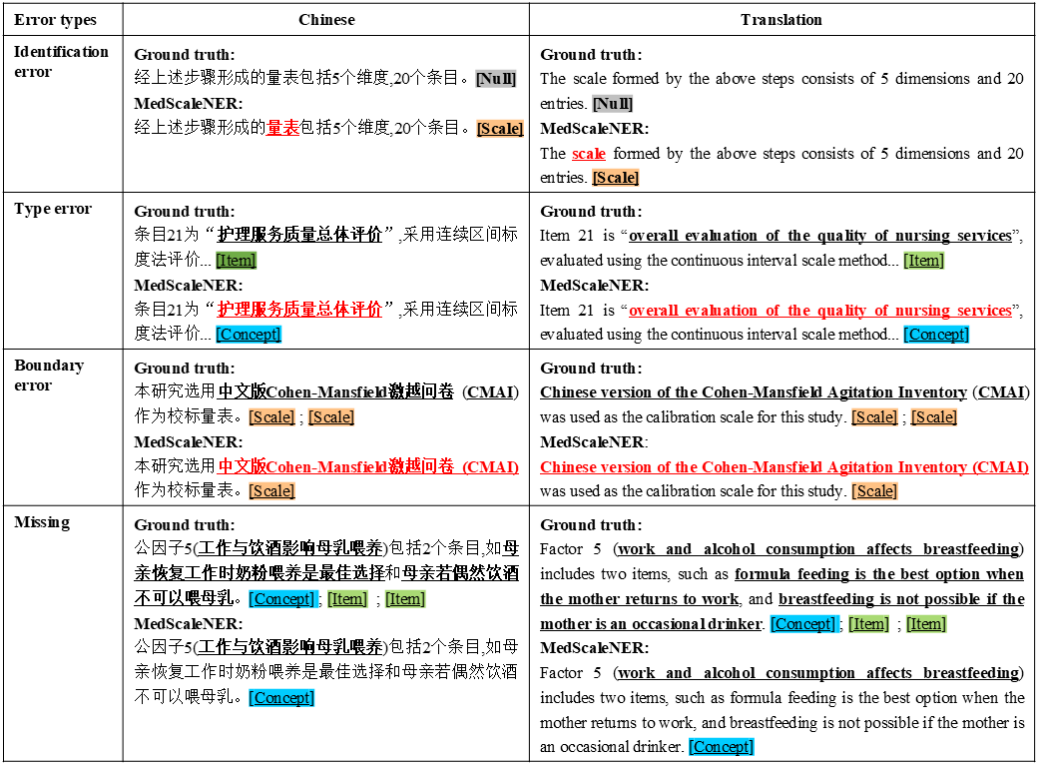
Examples of the four main error types identified in MedScaleNER: identification errors, type errors, boundary errors, and missing entities.

Figure 6 provides examples of each error type, illustrating the nature of these mistakes. Identification errors were the most common and often resulted from ambiguous entity definitions. For example, generic terms like “item” or “scale” were sometimes misinterpreted as specific entities due to their inclusion in prompt definitions. Type errors occurred when entities were recognized but misclassified. For instance, “overall evaluation of the quality of nursing services” was mistakenly labeled as a concept rather than an item. Boundary errors included incorrect inclusion or exclusion of surrounding text or punctuation, such as parentheses or modifiers that should not be part of the entity span. Finally, missing entities were frequently associated with English names or abbreviations of scales and items, especially in cases involving long or complex strings.

## Discussion

### Principal Findings

This study is among the first to explore the use of LLMs and prompt engineering for NER tasks related to Chinese medical scales. We proposed a novel prompt framework, MedScaleNER, which enhances the adaptive learning capabilities of LLMs by dynamically retrieving optimal examples through KNN retrieval. By using a CoT strategy, the framework decomposes the complex task of entity recognition into two sequential steps: first, identifying entity types and then labeling entities. This approach strengthens the logical reasoning ability of LLMs. Additionally, incorporating self-verification mechanisms ensures the accuracy of the final recognition results, improving the reliability of the model’s outputs.

Our evaluation of the self-constructed CMedS-NER dataset demonstrated that MedScaleNER effectively recognizes medical scale–related entities. The dataset, comprising 720 full-text Chinese academic papers with 27,499 annotated entities, is a high-quality resource for training and evaluating NER models in this specialized domain. Notably, in low-resource settings with as few as 205 sentences, MedScaleNER outperformed locally fine-tuned models such as BiLSTM-CRF (Chinese-BERT-wwm), W2NER (MacBERT), GLM-4-9B-Chat, and Qwen2-7B. When more annotated data became available (eg, 1023 sentences), MedScaleNER remained competitive. This low-resource performance is particularly significant in biomedical and clinical contexts, where domain-specific annotations are often expensive and time-consuming to produce.

Ablation studies further highlighted that KNN retrieval significantly improved performance in low-resource settings, aligning with previous findings [37] on the benefits of such strategies in ICL. Integrating CoT prompting and self-verification with KNN retrieval boosted the *F*_1_-score by approximately 6% when using 5% of the training data. This suggests that while retrieving representative examples is crucial, the structured CoT and self-verification steps are also important, contributing to more accurate and robust entity annotation than retrieval-based demonstration alone.

Although using high-quality demonstrations improved the LLM’s ability to recognize scale-related entities [25,45], performance declined when the number of examples exceeded an optimal threshold. Context length limitations, example ordering [46], and entity-type specific sensitivities influenced this trade-off. For instance, concept entities benefited from fewer examples compared to scale and item entities. It suggests that tailoring demonstration strategies by entity type could maximize performance.

Compared to traditional and fine-tuned NER methods designed for similar biomedical contexts, MedScaleNER offers several advantages. Conventional approaches often require extensive domain adaptation, large annotated corpora, or multiple rounds of fine-tuning to achieve competitive results [47,48]. In contrast, MedScaleNER excels under low-resource settings by leveraging KNN retrieval, CoT, and self-verification. Its flexible, task-oriented design allows simple modification of entity definitions to adapt to new domains, other languages, or even other LLM backbones. This adaptability supports broader generalizability, enabling MedScaleNER to scale beyond Chinese medical scales to other medical domains and even entirely different biomedical NER tasks.

Moreover, moving toward a human-centered medical scale NER workflow is crucial [23,49]. Allowing domain experts to provide feedback, customize prompt components, control retrieval parameters, and determine when to use self-verification can improve transparency, trust, and overall user satisfaction [50,51]. Such a human-in-the-loop approach ensures that MedScaleNER remains aligned with real-world clinical and research priorities, particularly important in dynamic health care environments.

### Limitations

Despite these strengths, there are limitations to this study. First, we primarily focused on the GLM-4 model, and future work should evaluate additional LLMs [52] such as LLaMA, Mistral, GPT, and PaLM, to validate generalizability. Second, our example retrieval strategy relied on KNN based on sentence similarity. Alternative retrieval strategies [53] and more advanced similarity models may further enhance performance. While we focused on three main scale-related entity types: scale names, measurement concepts, and measurement items, future research could extend this framework to other entities, such as functions, targets, and validity measures. Finally, integrating LLMs with traditional NER models could leverage the complementary strengths of both approaches, potentially resulting in more robust and accurate entity recognition systems.

### Conclusions

In this study, we introduced MedScaleNER, a task-oriented prompt framework that integrates demonstration retrieval, CoT prompting, and self-verification strategies to enhance the recognition of medical scale-related entities in Chinese medical literature. Evaluated on our self-constructed CMedS-NER dataset, MedScaleNER demonstrates robust performance even with limited annotated data. By allowing simple adjustments to prompt definitions, MedScaleNER readily adapts to diverse biomedical domains, languages, and entity types, making it a resource-efficient solution for broader information extraction challenges. This adaptability supports more efficient and reliable knowledge extraction, ultimately contributing to better clinical and research outcomes in MBC. By continuing to refine and expand MedScaleNER, we aim to advance automated knowledge extraction systems and promote the widespread adoption of MBC in health care.

## Appendix

Multimedia Appendix 1 Annotation scheme and guidelines.

Multimedia Appendix 2 Prompts for step 1.

Multimedia Appendix 3 Prompts for step 2.

Multimedia Appendix 4 Prompts for step 3.

Multimedia Appendix 5 Preliminary experimental results for optimal model selection.

Multimedia Appendix 6 Hyperparameters of locally fine-tuned models.

## Abbreviations

API: application programming interface
CMedS-NER: Chinese Medical Scale Corpus for Named Entity Recognition
CoT: chain-of-thought
ICL: in-context learning
KNN: k-nearest neighbor
LLM: large language model
MBC: measurement-based care
NER: named entity recognition

## Data Availability

The sample data and the source code are available on GitHub [54].
