# Supplementary material for: Prompt Framework for Extracting Scale-Related Knowledge Entities from Chinese Medical Literature: Development and Evaluation Study
Source: J Med Internet Res. 2025 Mar 18;27:e67033. doi: 10.2196/67033 (PMC11962316; doi:10.2196/67033)
Supplement: Multimedia Appendix 1 [file jmir_v27i1e67033_app1.docx]

Multimedia Appendix 1. Annotation scheme and guidelines.

| Annotation principle | Interpretation and example |
| --- | --- |
| Mention-level annotation | Identify and label specific words or phrases referring to particular entities in the text. |
| Language | Scale: Chinese and English mentions;  Concept: Chinese mentions only;  Item: Chinese mentions only; |
| Comprehensiveness | Entity mentions are labelled as they appear, temporarily disregarding the verbs that precede them to indicate research methodology, such as “delete”, “translate”, “revise”, etc. |
| Longest annotation principle | To guarantee the specificity of the entity mentioned. For example, “Common Module of Cancer Patients’ Life Quality Measurement Scale System” is labelled as a whole entity. |
| Type uniqueness | The same mention can only be labelled as one entity type. |
| *Types of non-labelling* |  |
| Nested entity | An entity cannot appear within another entity.  For example, “Common Module of Cancer Patient Life Quality Measurement Scale System” does not need to be labelled as “Common Module of Cancer Patient Life Quality Measurement Scale System” and “Cancer Patient Life Quality Measurement Scale”. |
| Discontinuous entities | Entities separated by other words or sentence elements in the text are not labelled. |
| Indicator pronouns | For example , “the scale”, “its”, “the factor” |
| Entity code | Concepts and items are often referred to in journal articles by codes, which are often not semantically distinct when used alone and are not labelled. For example, “Factor 1”, “Item 2”. |
