# Supplementary material for: Prompt Framework for Extracting Scale-Related Knowledge Entities from Chinese Medical Literature: Development and Evaluation Study
Source: J Med Internet Res. 2025 Mar 18;27:e67033. doi: 10.2196/67033 (PMC11962316; doi:10.2196/67033)
Supplement: Multimedia Appendix 2 [file jmir_v27i1e67033_app2.docx]

Multimedia Appendix 2. Prompts for Step 1

| Step1 | Prompt in Chinese | Translation |
| --- | --- | --- |
|  | 你是一个优秀的语言学家和命名实体识别专家。你的任务是根据给定的文本和实体类型列表{“量表”，“测量概念”，“测量项目”}，识别并列出文本中存在的实体类型。 | You are a sophisticated linguist and named entity recognition expert. Your task is to identify and list the entity types from the given entity type list: {“Scale”, “Concept”, “Item”}. |
|  | 以下是实体类型及其特征的详细说明：  0.“量表”：一种测量工具，通常以“***评分表”、“***量表”、“***问卷”、“***问题模块”等形式命名。常见命名形式为词或短语。  1.“测量概念”：用来评估特定测量概念的单个问题、陈述或任务描述。一般通过单个简单名词、短语、陈述句、问句等形式呈现，常被双引号包围。  2.“测量概念”：量表中的理论变量，有“**维度”、“**领域”、“**方面”、“**成分”、“**次领域”、“**子领域”、“**变量”、“**因子”、“**因素”等不同称谓。 | The following are the definitions of the entity types and their characteristics:  0. “Scale”: a measurement tool, usually called a “rating scale”, “questionnaire” or “question module”. It is often named using words or phrases.  1. “Concept”: the variables in the scale, such as “dimension”, “domain”, “aspect”, “sub-domains”, “variables”, “factor” and other different designations.  2. “Item”: A single question, statement, or task used to assess a particular measurement concept. They are usually presented as simple nouns, phrases, sentences, questions, etc. |
|  | 按如下json格式进行结果输出:{实体类型1,实体类型2}。请注意:  1.只需要输出结果,不要输出其他内容。  2.所有输出的实体类型必须在实体类型列表中,如果句子中不存在任何实体类型,则输出{null}。  3.不要重复输出结果列表中已经存在的实体类型。 | Output format: {entity type 1, entity type 2,…}.  1. Only output results.  2. The entity types must be in the list. If no entity types in the sentence, output {null}.  3. Don’t repeat existing entity types. |
|  | 输入：“句子”  输出：{“…”} | Input: “Sentence”  Output：{“…”} |
