# Supplementary material for: Prompt Framework for Extracting Scale-Related Knowledge Entities from Chinese Medical Literature: Development and Evaluation Study
Source: J Med Internet Res. 2025 Mar 18;27:e67033. doi: 10.2196/67033 (PMC11962316; doi:10.2196/67033)
Supplement: Multimedia Appendix 3 [file jmir_v27i1e67033_app3.docx]

Multimedia Appendix 3. Prompts for Step 2

| Step2 | Prompt in Chinese | Translation |
| --- | --- | --- |
|  | 你是一个优秀的语言学家和命名实体识别专家。你的任务是对给定文本中的“量表”，“测量概念”相应实体进行标记。 | You are a sophisticated linguist and named entity recognition expert. Your task is to label the entities that match the entity types listed in output 1. |
|  | 为帮助你理解，下面给出这k类实体的详细说明： | The following are the definitions of the entity types and their characteristics: |
|  | 标记规则如下：  1.如果存在“实体类型”实体，在每个实体的左侧和右侧分别添加<实体类型>和</实体类型>标记符号。  3.如果给定文本中不存在“实体类型”，则直接输出原始文本。  按如下json格式进行结果输出：{“标记结果”：“标记后的句子”}。请注意：只输出结果，不要输出其他内容。 | The rules for labelling are as follows:  1. Add the <entity type> and </entity type> markers to both sides of each entity.  2. If no entity types in the text, then directly output the original text.  Output the result in the following json format: {Output: “Marked sentence”}. Please note: only output the result, not other contents. |
|  | 下面是k个实例:  输入：“句子”  输出：{“标记后的句子”} | Here are k examples:  Input: “Sentence”  Output：{“ Marked sentence ”} |
|  | 输入：“句子”  输出：{“…”} | Input: “Sentence”  Output：{“…”} |
