# Supplementary material for: Prompt Framework for Extracting Scale-Related Knowledge Entities from Chinese Medical Literature: Development and Evaluation Study
Source: J Med Internet Res. 2025 Mar 18;27:e67033. doi: 10.2196/67033 (PMC11962316; doi:10.2196/67033)
Supplement: Multimedia Appendix 4 [file jmir_v27i1e67033_app4.docx]

Multimedia Appendix 4. Prompts for Step 3.

| Step3 | Prompt in Chinese | Translation |
| --- | --- | --- |
|  | 你是一个优秀的语言学家和命名实体识别专家。请阅读给定的语境文本，据此复核已标记实体的类型是否正确。注意:只输出验证结果，不要输出其他内容。  下面是k个实例: | You are a sophisticated linguist and named entity recognition expert. Verify whether the entity labelling markers in output 2 are accurate. Only display the validation results.  Here are k examples: |
|  | **输入**：  {“语境文本”：“…”,  “待验证实体及其类型”：  [{“entity 0”:”…”, “type”:”…”};  {“entity 1”:”…”, “type”:”…”};  …]}  输出：  {[“entity 0”:” …, “type”: “…”, “result”: 是];  [“entity 1”:” …, “type”: “…”, “result”: 否];  …} | Input:  {“Sentence”: “…”,  “Entity mention”:  [{“entity 0”:”…”, “type”:”…”};  {“entity 1”:”…”, “type”:”…”};  …]}  Output:  {[“entity 0”:” …, “type”: “…”, “result”: Yes];  [“entity 1”:” …, “type”: “…”, “result”: No];  …} |
