# Supplementary material for: Prompt Framework for Extracting Scale-Related Knowledge Entities from Chinese Medical Literature: Development and Evaluation Study
Source: J Med Internet Res. 2025 Mar 18;27:e67033. doi: 10.2196/67033 (PMC11962316; doi:10.2196/67033)
Supplement: Multimedia Appendix 5 [file jmir_v27i1e67033_app5.docx]

Multimedia Appendix 5. Preliminary experimental results for optimal model selection. This preliminary experiment was conducted on a simplified dataset, where each sentence contained at least one of the ten predefined scale entities. This setup focused solely on recall, aiming to evaluate the LLMs’ capacity to detect known entities in a simple scenario.

| Model | TP(%) | FN(%) |
| --- | --- | --- |
| GPT-3.5-turbo | 10 | 90 |
| GLM-4-0520 | 90 | 10 |
| ERNIE-Bot-turbo | 70 | 30 |
| Moonshot-v1-8k | 70 | 30 |
| AGI Sky-Chat-3.0 | 60 | 40 |
| Qwen-turbo-0624 | 80 | 20 |
