# Supplementary material for: Prompt Framework for Extracting Scale-Related Knowledge Entities from Chinese Medical Literature: Development and Evaluation Study
Source: J Med Internet Res. 2025 Mar 18;27:e67033. doi: 10.2196/67033 (PMC11962316; doi:10.2196/67033)
Supplement: Multimedia Appendix 6 [file jmir_v27i1e67033_app6.docx]

| Hyperparameter | BiLSTM-CRF  (Chinese-BERT-wwm) | W2NER  (MacBERT) | GLM-4-9B-Chat  （128K） | Qwen2-7B  （128K） |
| --- | --- | --- | --- | --- |
| Max_seq_len | 512 | 512 | 2048 | 2048 |
| Learning_rate | 5e-5 | 5e-5 | 5e-5 | 5e-5 |
| Batch_size | 50 | 50 | 4 | 4 |
| Epoch | 50 | 50 | 5 | 5 |
| Optimizer | Adam | Adam | Adam | Adam |
| RNN_hidden_size | 256 | 500 | 500 | 500 |
| Finetunig_type | - | - | Lora | Lora |
| Lora_rank | - | - | 8 | 8 |
| Lora_dropout | - | - | 0.1 | 0.1 |
